# Learning an EMG Controlled Game: Task-Specific Adaptations and Transfer

**DOI:** 10.1371/journal.pone.0160817

**Published:** 2016-08-24

**Authors:** Ludger van Dijk, Corry K. van der Sluis, Hylke W. van Dijk, Raoul M. Bongers

**Affiliations:** 1 University of Groningen, University Medical Center Groningen, Center for Human Movement Science, Groningen, The Netherlands; 2 University of Groningen, University Medical Center Groningen, Department of Rehabilitation Medicine, Groningen, The Netherlands; 3 NHL University of Applied Sciences,Faculty of Engineering, Serious Gaming Group, Leeuwarden, The Netherlands; Shanghai Jiao Tong University, CHINA

## Abstract

Video games that aim to improve myoelectric control (myogames) are gaining popularity and are often part of the rehabilitation process following an upper limb amputation. However, direct evidence for their effect on prosthetic skill is limited. This study aimed to determine whether and how myogaming improves EMG control and whether performance improvements transfer to a prosthesis-simulator task. Able-bodied right-handed participants (N = 28) were randomly assigned to 1 of 2 groups. The intervention group was trained to control a video game (Breakout-EMG) using the myosignals of wrist flexors and extensors. Controls played a regular Mario computer game. Both groups trained 20 minutes a day for 4 consecutive days. Before and after training, two tests were conducted: one level of the Breakout-EMG game, and grasping objects with a prosthesis-simulator. Results showed a larger increase of in-game accuracy for the Breakout-EMG group than for controls. The Breakout-EMG group moreover showed increased adaptation of the EMG signal to the game. No differences were found in using a prosthesis-simulator. This study demonstrated that myogames lead to task-specific myocontrol skills. Transfer to a prosthesis task is therefore far from easy. We discuss several implications for future myogame designs.

## Introduction

Although video games that aim to improve myoelectric control are becoming an important part of the rehabilitation process following an upper limb amputation [1,2], little is known about the benefits of training myoelectric control by video gaming (i.e. using a myogame). Many studies so far limit their research to the development of the myogame, and do not include an evaluation of training effects after using the game [3,4]. Studies that did include the training of the myogame often did not provide statistical support for apparent improvement in performance and, with the exception of one study [5], none have used a control group [6–11]. Most importantly, there is, to our knowledge, only one study that determined whether training effects will subsequently transfer to other myoelectric tasks, such as the use of a prosthesis [12]. That study showed a task-specific learning effect that transferred only on a few highly task-specific outcome measures. The study thus raised the concern that the way myogames are currently adopted in clinical situations might not promote any transfer of skill. The current paper aims to evaluate this implication.

By focusing research on motivational aspects and playability rather than on explicitly designing for transfer to activities of daily living (ADL) (see e.g. [6,9]), myogame development has been able to proceed without paying much attention to aspects that could constrain transfer to actual prosthesis use. For example, studies often do not attempt to simulate the way the amplitude of the myosignal is related to the opening and closing of the prosthesis hand [6,7]. Other technical constraints of the prosthesis are also not taken into account (e.g. motor delays, EMG response curves)–nor can they be, as detailed technical specifications are often not supplied by prosthesis manufacturers. Finally, the consensus in motor learning literature is that training is task-specific–that is, in order to transfer a skill between tasks, the goal of these tasks should be as similar as possible [13–16]. So far however, myogames deliberately deviate from real ADL tasks in order to remain entertaining and motivating [1,3–10].

These concerns led Van Dijk et al. [12] to create an experimental set-up that controlled both technical constraints and the amount of task similarity. After providing evidence that the used myogames were actually learned, the study showed to what extent the learning of these games affected the performance of a prosthesis task. The results showed that neither the technical similarity in EMG interfacing nor the goal of the gaming task will ensure transfer. Rather, only the training conditions in which very specific feedback was added to the game elicited transfer to the use of a prosthetic device. Crucially, this feedback was not only relevant to attaining the gaming task, but the feedback was also important to the prosthetics grasping task that the participants needed to perform to assess transfer.

Since the current generation of myogames typically has little similarity with the activities in daily life they set out to promote, the question therefore becomes to what extent the set-up currently adopted in serious gaming research will elicit transfer to a basic prosthesis task. The main aim of this study is to determine what learning and transfer effects can be expected from training with the current generation of myogames. We aimed to stay as close as possible to currently established practices: we developed a basic but motivational myogame that is comparable to those currently used in research, and we used a prosthesis task similar to the previous transfer study that reflects the basic settings and function used by patients in ADL [12].

To reach our aim this study answers three questions. A prerequisite for showing transfer is showing learning during training. Therefore, the first question is whether our serious game that incorporates a myoelectric control interface can be learned. If the myogame is learned we expect an increase in accuracy of in-game performance after training in comparison to the sham training. Finding a learning effect, the second question is what change in the gaming task might account for this. Although the above mentioned study [12] does not report on this issue, it suggests highly task-specific adaptations of the myosignal. We evaluate this prediction by looking into the relation between the myosignal and the goal of our game (i.e. intercepting a ball). The third and final question is whether learning effects of this myogame transfer to the actual use of a prosthesis during a grasping task. If so, we expect participants to get more skilled at using a myoelectrically controlled prosthesis. This skill improvement will be reflected in (a) the participants’ ability to adapt the aperture of the grasping hand to the size of an object [12]. This adjustment has been found in experienced prosthesis users [17] and is also typical for grasping with an intact hand [18–21]. Skill improvement will also be reflected in (b) the time that the myoelectric hand remains maximally opened: this is expected to be shorter in skilled prosthetic users [22].

## Methods

### Participants

Twenty-eight able bodied adults participated (mean age 21.39 (SD 1.95) y); 21 men and 7 women. The participants played video games for 4.32 (SD 4.38) hours a week. All participants (1) were right handed, (2) had corrected to normal vision, (3) were free of any (history of) disorders of the arms or upper body, and (4) had no prior experience in the use of myoelectric devices. The study was approved by the local ethics committee (Ethics Committee for Human Movement Sciences, University of Groningen, the Netherlands) and a signed informed consent was obtained from all participants prior to the start of the experiment. Upon completion of the experiment all participants received a gift voucher.

### Materials

In order to train the use of myoelectric control in a serious game, a customized version of the game “Breakout” was created (originally created by Atari Inc.). This game, called “Breakout-EMG” was run on a laptop computer. Two active socket 13E200 MyoBock electrodes (Otto Bock Healthcare products, Austria) were used. The electrodes used a bandwidth of 90-480Hz and a notch filter at 50Hz. After that the signal was rectified and low pass filtered (2nd-order). The amplification of the signal could be controlled linearly with a gain controller. These signals were fed into the laptop computer, via a NI-USB 6009 data acquisition device (National Instruments Corporation, USA) that sampled the signals at 125 Hz. Custom LabView software (National Instruments Corporation, USA) digitally filtered the signals (low pass filter, cutoff frequency 150 Hz). The game sampled these digitally filtered EMG signals at 50 Hz. To log all the gaming data for analyses, in a separate process the (x and y) positions of the elements of the game were written to a text file at 90 Hz.

As a sham training, a standard platform game called “Super Mario Bros” was run on a Nintendo Entertainment System (Nintendo Co. Ltd, Japan). This game was connected to a LCD-TV monitor.

**Fig 1:**
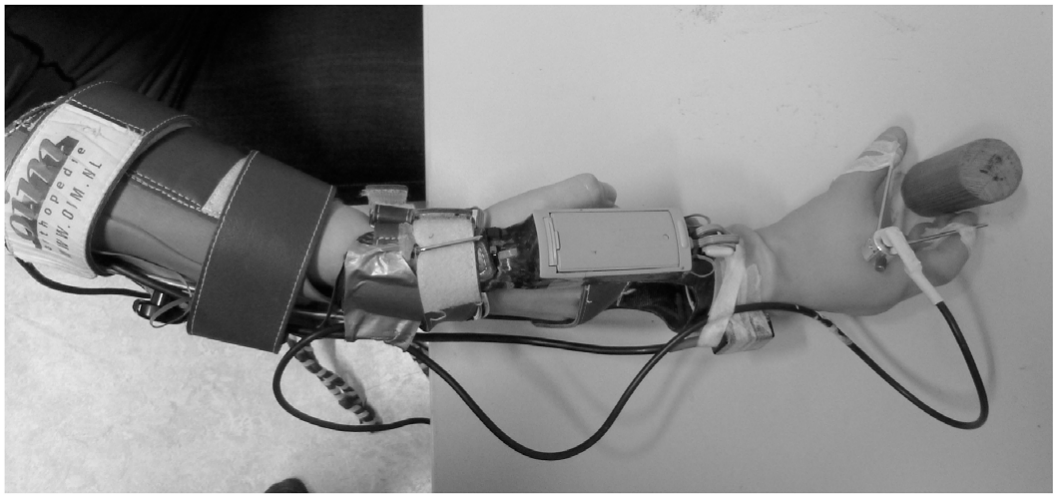
The myoelectric simulator. Top view of the myoelectric simulator while grasping the medium cylinder. The goniometer is attached to the thumb and index finger.

To resemble a myoelectric upper-extremity prosthesis for a transradial amputation level as closely as possible, a myoelectric simulator was developed (Fig 1) [12,22–24].

This simulator consisted of a myoelectric hand attached to an open cast in which the hand could be placed, and a splint that was adjustable in length and attached the simulator to the forearm with a Velcro sleeve. The myoelectric hand was a MyoHand VariPlus Speed (Otto Bock Healthcare products, Austria) with proportional speed (15–300mm/s) and grip force control (0-~100N).

During the myoelectric simulator task three wooden cylinders were grasped. These cylinders were 10 cm in height and were either 2 cm (small), 4 cm (medium) or 6 cm (large) in diameter. In order to measure the aperture of the myoelectric hand during the grasp, a goniometer (Cermet PC300 potentiometer, Contelec, Switzerland) was attached to the thumb and index finger of the hand. The goniometer sampled the angle of the hand at 2000Hz and sent this data to the laptop computer. Because of a technical problem the angular data on the trials grabbing the largest cylinder could not be established. Therefore, only the data on grabbing the small and medium cylinders are presented.

### Design

**Fig 2:**
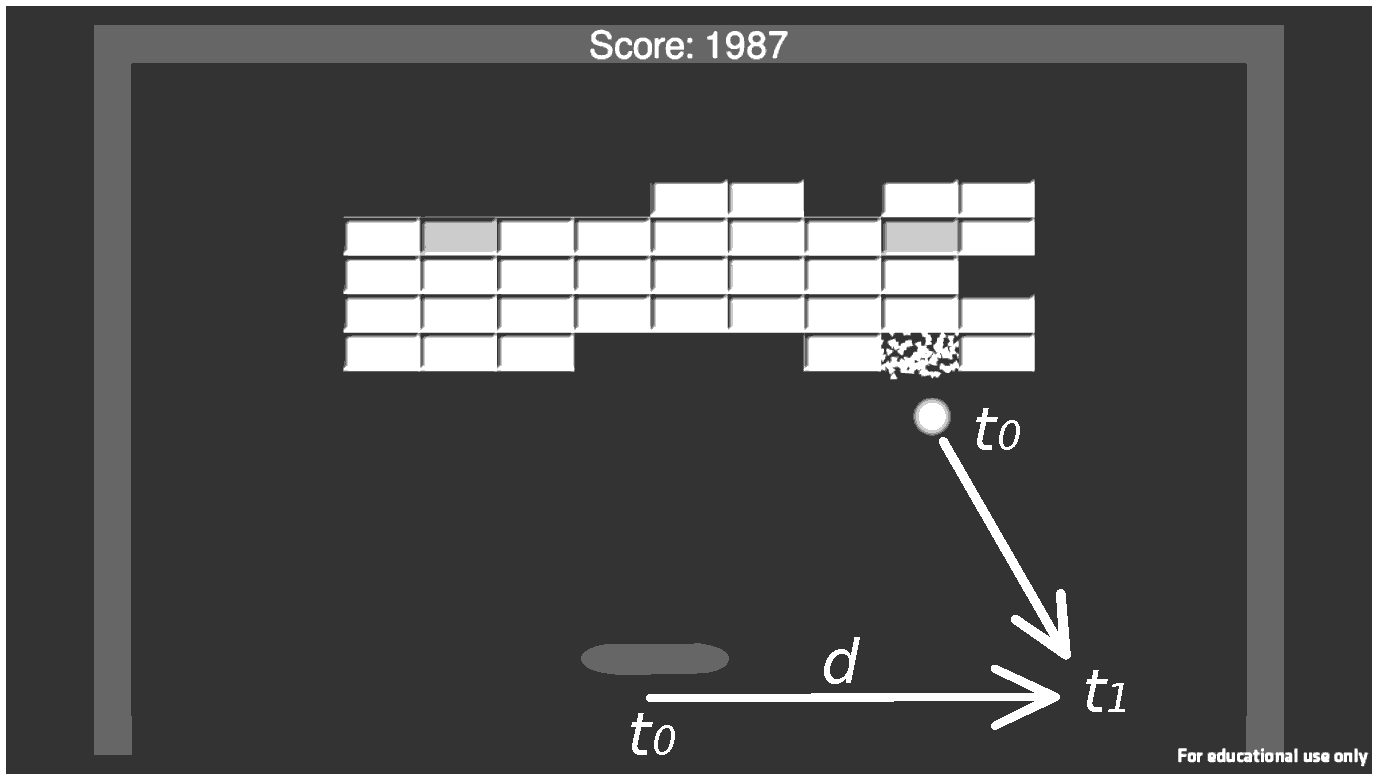
Breakout-EMG. Screenshot of Breakout-EMG showing an example of a terminal ball drop. The distance that the ball needed to move was calculated by determining the interval from the point at which the ball began to drop down towards the ground (t0) and the point at which the ball got to the height (y-position) of the paddle (t1). The required distance was the difference in position of the paddle at t0 and the position of the ball at t1. The required distance was correlated to the observed net EMG signal (see text for details).

Participants were randomly assigned to either the Breakout-EMG group (n = 16) or to the Control group (n = 12) as they signed up. The Breakout-EMG group trained the game “Breakout-EMG” (see Fig 2). Breakout-EMG was a videogame in which the objective was to intercept a bouncing ball so that it did not hit the ground. By bouncing the ball with the paddle, a wall of blocks could be hit. The overall objective of the game was to clear the screen of these blocks. The movement of the paddle to the left and right was controlled using the myoelectric signals from the flexor or extensor muscles of the wrist, respectively. The speed of the paddle was proportional to the amplitude of the EMG signals. During testing and training with Breakout-EMG the participants were free to hold their arm in any position they felt comfortable with as long as the electrodes were not perturbed (e.g. by hitting the table). The Control group trained in playing Super Mario Bros. In this game the objective was to control an avatar and safely guide him through a world by jumping platforms and avoid enemies. The game was played using a standard hand held Nintendo controller, which was held in the palms of both hands and typically operated using both thumbs (i.e. pressing down with the left thumb for moving the avatar left and right, and pressing down with the right thumb for jumping). The experiment was conducted in 5 days and consisted of 4 training sessions. On the first day a pretest was performed, after which 4 days of training followed. On the fifth day a posttest was performed. For practical reasons, participants were randomly assigned to either have the first training session after the pretest on day 1, or have their fourth training session prior to the posttest on day 5.

### Procedure

#### Fitting of the electrodes

Prior to playing Breakout-EMG, the electrodes were fitted by palpating for the most prominent muscle bellies of the extensors and flexors of the wrist during contraction. The electrodes were subsequently placed at those sites and held in place by a flexible wristband. The signals were filtered and sent to the game computer. In the game environment both signals were calibrated by determining the minimum and maximum value of each electrode independently and scaling each signal to a standard range before the game began. The signal was amplified so that reaching the maximum movement speed in the game required 20% of the maximum voluntary contraction (MVC) of the muscles. This was necessary to allow for comfortable game-play and prevented muscle fatigue during training.

For controlling the hand of the prosthesis simulator at the pre- and posttest, the sites for fitting the electrodes were similarly determined. The electrodes were subsequently placed by attaching the prosthesis simulator to the participant's arm. The sensitivity of the electrodes was adjusted to the upper threshold for each participant individually, so that the maximum EMG signal that could be sustained for 2 seconds of each participant corresponded to the maximum opening and closing speed of the myoelectric hand.

#### Pretest and posttest

The pretest was equal to the posttest and these tests were used to determine the improvement in skill in playing Breakout-EMG and in using the prosthesis simulator. To determine whether the myogame Breakout-EMG could be learned, during the pretest and the posttest, participants from both the Breakout-EMG group and from the Control group were asked to play one level of the game. This level (level 1) consisted of a screen with 45 blocks that needed to be hit by intercepting and bouncing a ball (see Fig 2). The level started when the experimenter pressed start and finished when the last block was hit. The participants did not receive specific instructions other than to play the game.

In order to find out whether any improvement of skill in Breakout-EMG transferred to using the prosthesis simulator, the change in performance during a simple grasping task was measured. In this task participants sat at a comfortable position in front of a table wearing the prosthesis simulator. Prior to the start of the task participants were instructed to maximally open and close the myoelectric hand to establish the minimum and maximum aperture for each participant. Starting with a closed myoelectric hand, they were then asked to grasp one of three wooden cylinders that were placed at 35 cm from the starting position of the myoelectric hand, lift the cylinder slightly, and then place it back at its original position. Each cylinder needed to be grasped five times. The order in which the cylinders were presented was randomized. The participants were instructed to be as accurately as possible in grasping, emphasizing not to focus on speed of performance but rather to focus on not dropping the cylinder while grasping.

#### Training sessions

In each session the Breakout-EMG group trained by playing Breakout-EMG for 20 minutes. To keep the participants challenged during training, the game consisted of three levels. These levels differed in the amount of blocks to be hit (increasing the difficulty of attaining a high accuracy–i.e. a perfect score). After completion of each level, the participants received feedback on their performance: on their accuracy in intercepting the ball, on the number of points scored (with each block hit points were added) and the duration of the level. After playing all three levels, the participants started again at level 1. There were no negative consequences to a bad performance. The game had no sound.

The Control group played Super Mario Bros for 20 minutes per session. The participants were instructed to only play the first four levels of the game (i.e. level 1–1 to 1–4) and then start over. To match Breakout-EMG training, this game was muted so that it too had no sound.

### Data analysis

Using customized Matlab (The Mathworks Inc., USA) scripts, all dependent variables used to determine in-game performance were calculated from the output file provided by Breakout-EMG (all dependent variables are listed in Table 1). As playing Breakout-EMG proficiently required a high degree of accuracy in intercepting the ball, we looked at accuracy for in-game learning effects. The accuracy with which the ball was intercepted was determined as the number of balls intercepted divided by the total number of balls that dropped to the ground level.

**Table 1:** Dependent variables. All dependent variables and their within subjects factors for the repeated measures ANOVA. The within subjects factor “Test” had two levels: the pretest and the posttest. The within subjects factor “Cylinder” had two levels: the small and the medium cylinder. The between subjects factor for all variables was “Group,” which had two levels: the Breakout-EMG and the Control group. All combinations of interactions, both between the within subjects factors and between the within and between subjects factors, were also tested for effects (see text for details).

	Variable	Within subjects factor(s)	Between subjects factor
**In-game performance**	Accuracy	Test	Group
	EMG-ball coupling	Test	Group
**Transfer to prosthesis**	Maximum hand opening	Test and Cylinder	Group
	Standard deviation of the maximum hand opening	Test and Cylinder	Group
	Plateau phase	Test and Cylinder	Group

To look into specific adaptations of the EMG signal to the goal of the game, we defined the “EMG-ball coupling” as a measure of adaption. The strength of the EMG-ball coupling was determined by calculating the correlation between the distance the paddle needed to move and the observed net EMG signal during the terminal drop of the ball. We calculated the required distance (and direction) that the paddle needed to travel from the start to the end of each terminal ball drop (see Fig 2). The start of the terminal ball drop was defined as the point in time where the last change in direction of the ball occurred before reaching the height of the paddle. A change in direction less than 1 cm from the height of the paddle was disregarded, as this change hardly influenced the required position of the paddle to intercept. The net EMG signal was the integral of the difference between the calibrated EMG signal of the flexor and extensor muscle, over the duration of the drop of the ball. The net EMG signal thus had both a magnitude and a direction, which corresponded to the speed the EMG signals gave to the paddle.

Changes in the use of the myoelectric simulator were determined from the angular data from the goniometer using customized Matlab scripts. The angular data was filtered using a low pass filter (cutoff frequency 20 Hz). Subsequently, the start and end of the opening as well as of the closing of the myoelectric hand were determined from the data. If participants were better able to control the prosthesis simulator due to a more controlled use of EMG signal in the game, we expect participants to better adjust the hand opening to the size of the cylinder; requiring a smaller maximum hand opening (MHO) during the plateau phase as they learned to use the prosthesis [12,17–21]. The plateau phase was defined as the time from the end of the opening of the hand to the start of the closing, and by definition contained the maximum hand opening. Based on previous research [22], we also expect that increased prosthetic skills would show as a shorter plateau phase. As the goniometer was sometimes repositioned between participants and sessions, we normalized the angular data to a value between 0 and 1 based on the measured minimum and maximum value of each participant prior to analysis. The maximum hand aperture corresponds to a distance between the thumb and index finger of about 10 cm. So a change in aperture of 0.1 corresponds to ~1 cm in change in distance between thumb and index finger.

To determine learning effects, several repeated measures ANOVA's were conducted on accuracy and on the strength of the EMG-ball coupling, with test (pretest, posttest) as a within subjects factor and group (Breakout-EMG, Control) as a between subjects factor. To determine transfer, repeated measures ANOVA's were conducted on the maximum hand opening, on the duration of the plateau phase, and on the standard deviation of the maximum hand opening, with test (pretest, posttest) and cylinder (small, medium) as a within subjects factor and group (Breakout-EMG, Control) as a between subjects factor. A summary of all planned analyses can be found in Table 1.

Based on their skewness and on a Shapiro-Wilk test for normality, we checked the normality of the dependent variables. All variables were judged to be normally distributed, with the exception of the MHO and the SD-MHO. We therefore transformed the data on these variables using a square root transformation (x_trans_ = √((x_max_ + 1) + x). As a precautionary measure we repeated this procedure for the EMG-ball coupling. To check the effects of the distribution we then repeated our analyses of the MHO, the SD-MHO and the EMG-ball coupling on the pre- and posttest with these transformed data. None of the analyses on the transformed data differed from the analyses on the non-transformed data. We therefore present only the results on the non-transformed data here.

Effect sizes were calculated using generalized eta-squared (*η*^*2*^_*G*_) [25,26]. For the in-game learning effects, the Breakout-EMG group is expected to improve relative to controls. Therefore follow up comparisons were done using one-tailed independent t-tests (with Bonferroni correction for multiple comparisons). All analyses used a significance level of *α* = .05.

## Results

### In-game performance

**Fig 3:**
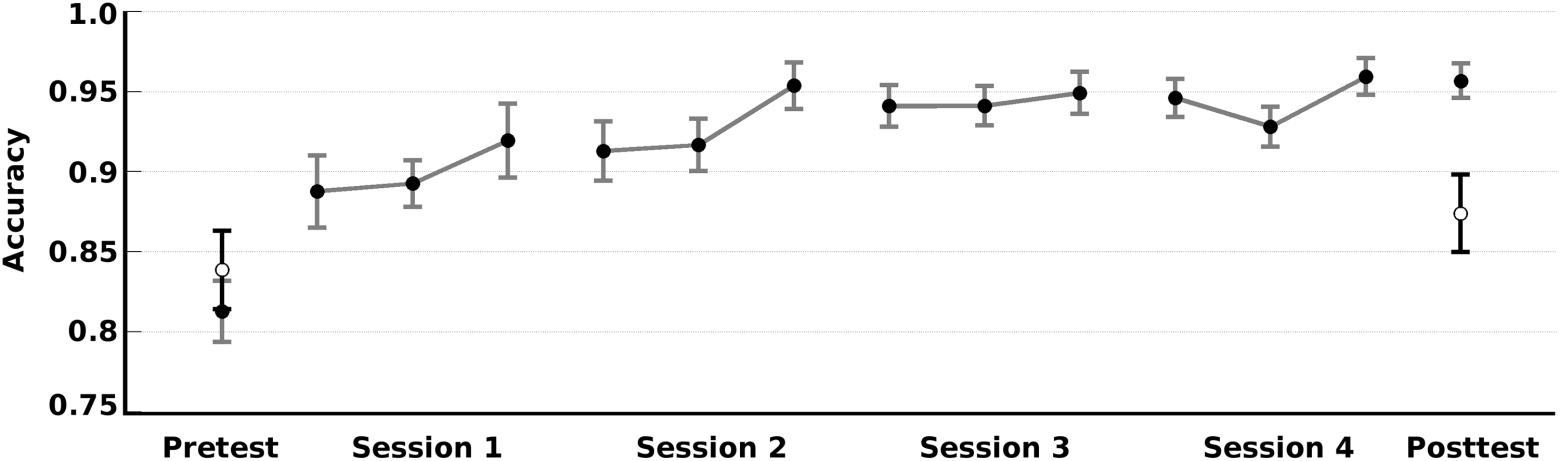
In-game accuracy. Mean accuracy (and standard error of the mean) on both the pretest and the posttest of the Breakout-EMG group (black points with grey error bars) and of the Control group (white points with black error bars), as well as the accuracy on all sessions for the Breakout-EMG group only. Each point denotes one trial of playing level 1 of the game. During the training sessions, after completing level 1 the participants played a trial at level 2 and a trial at level 3 before having another trial playing level 1. The accuracy on levels 2 and 3 are not shown. The number of trials participants played during a session depended on the time participants required to complete each trial. Therefore, not all participants managed to play three trials at level 1. The data on the first trial of each session is based on all 16 participants. The data on the second trial of each session is based on 15–16 participants. The third trial of each session is based on the data of 11–14 participants. Note that the biggest improvement occurred from pretest to the first session. There was a significant test effect from pre- to posttest. However, the improvement in the Breakout-EMG group was significantly greater than Controls (see text for details).

The accuracy of the Breakout-EMG group across all sessions, and the accuracy of the Control group on the pre- and posttest can be found in Fig 3. The increase in accuracy appeared to have been greatest at the start of the training. The improvement in accuracy after all training sessions was compared to the Control group. Accuracy of both the Breakout-EMG group and the Control group improved from pretest to posttest.

Importantly, the increase in accuracy after all training sessions was significantly greater for the Breakout-EMG group. A repeated measures ANOVA revealed a strong main effect for Test (*F*(1,26) = 58.25, *p* < .001, *η*^*2*^_*G*_ = .55), and a significant interaction effect Test x Group: *F*(1,26) = 21.39, *p* < .001, *η*^*2*^_*G*_ = .20. A follow up analysis revealed this improvement was explained by a difference between groups on the posttest (*t*(26) = -3.42, *p* = .002).

**Fig 4:**
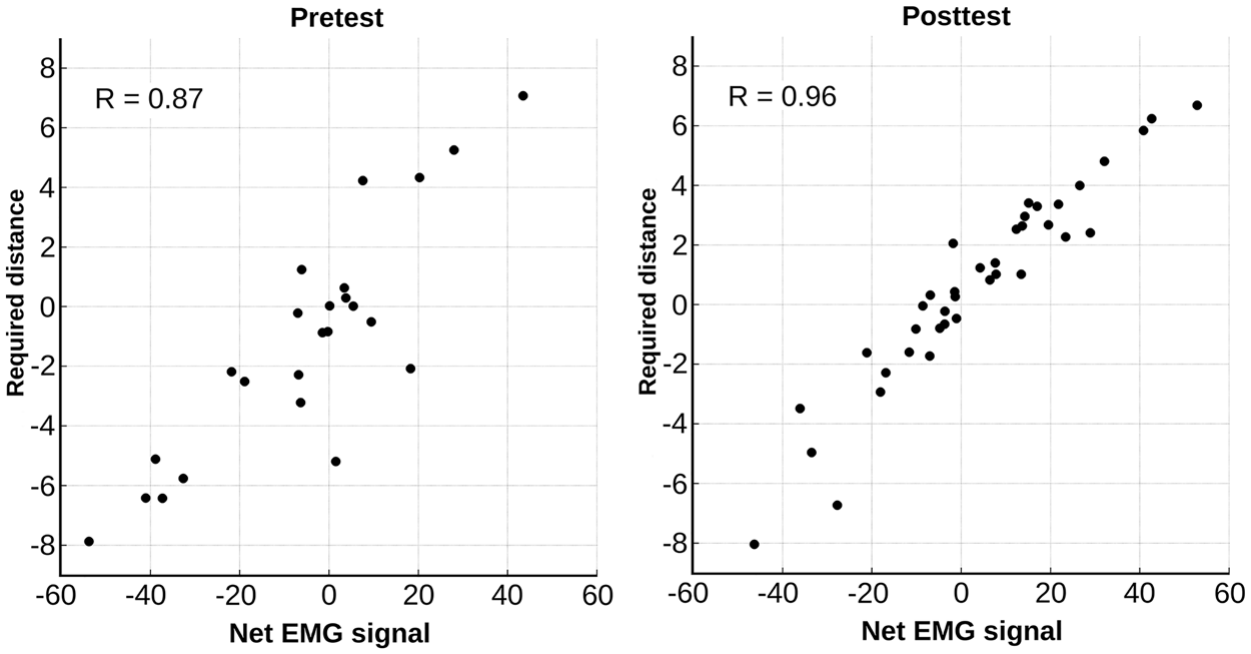
EMG-ball coupling. Representative example of the relation between the net EMG signal (x-axis) and the required distance (cm) to intercept the ball (y-axis) during a full trial of Breakout-EMG (Note that distance is actually expressed in units specific to the program used to design the game. However, on the monitor we used, these units are approximately equivalent to centimeters.). The net EMG signal has no unit of measurement but is the integral of the difference between the flexor and extensor EMG signal over the duration of the ball drop. Each point represents one interception attempt. To the left an example of a pretest, to the right an example of the posttest (of the Breakout-EMG group).

**Fig 5:**
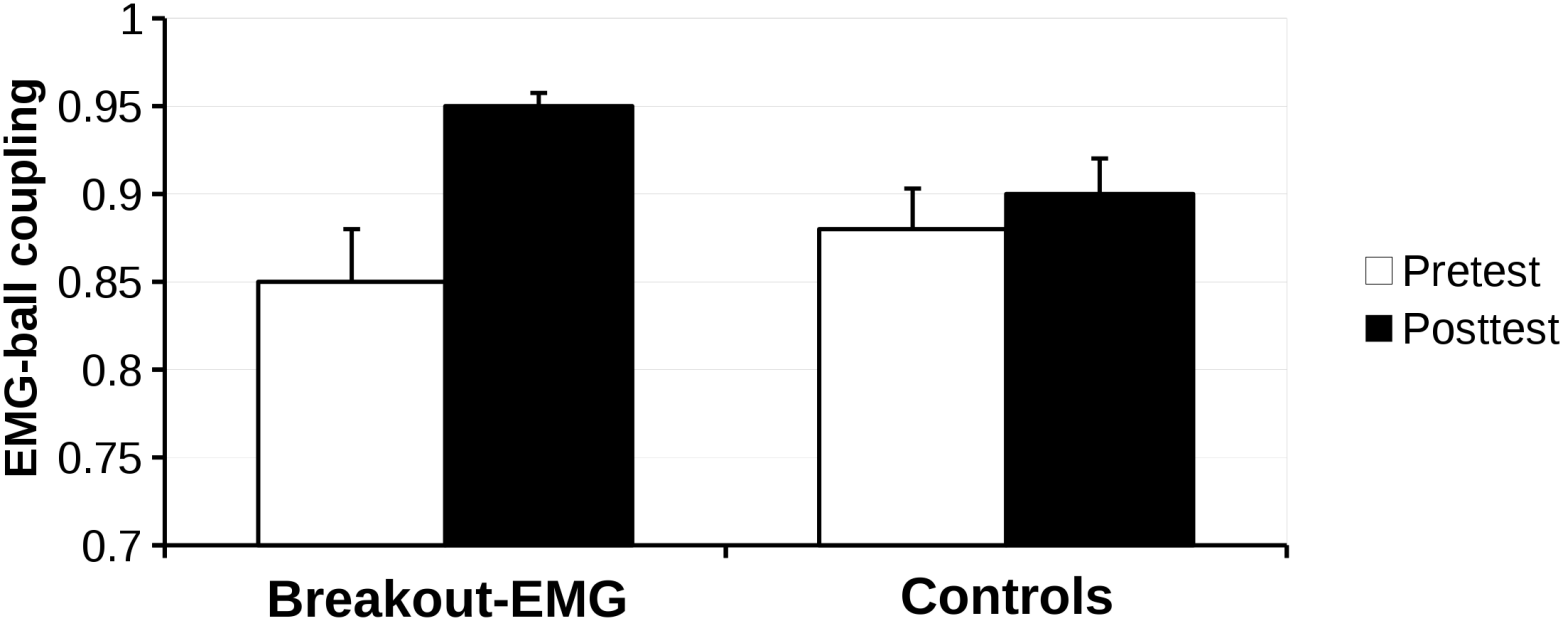
Change in EMG-coupling. Mean strength of the EMG-ball coupling (and standard error of the mean) on the pretest and the posttest for both groups. Both groups showed a significant test effect. However, the improvement in the Breakout-EMG group was significantly greater than Controls (see text for further details).

To better understand the changes in performance of the game, we examined the goal specific adaptation of the EMG signal from pretest to posttest. For this we used the strength of the EMG-ball coupling. An example of the EMG-ball coupling on a typical pretest and posttest is shown in Fig 4. The effect of the training on the EMG-ball coupling can be found in Fig 5.

A repeated measures ANOVA on the strength of the EMG-ball coupling revealed a main effect for Test (*F*(1,26) = 10.09, *p* = .004, *η*^*2*^_*G*_ = .25), and a significant interaction effect Test x Group: *F*(1,26) = 4.76, *p* = .038, *η*^*2*^_*G*_ = .12. A follow up analysis revealed this improvement was due to a difference between groups on the posttest (*t*(14.46) = -2.42, *p* = .029).

### Transfer to prosthesis use

**Fig 6:**
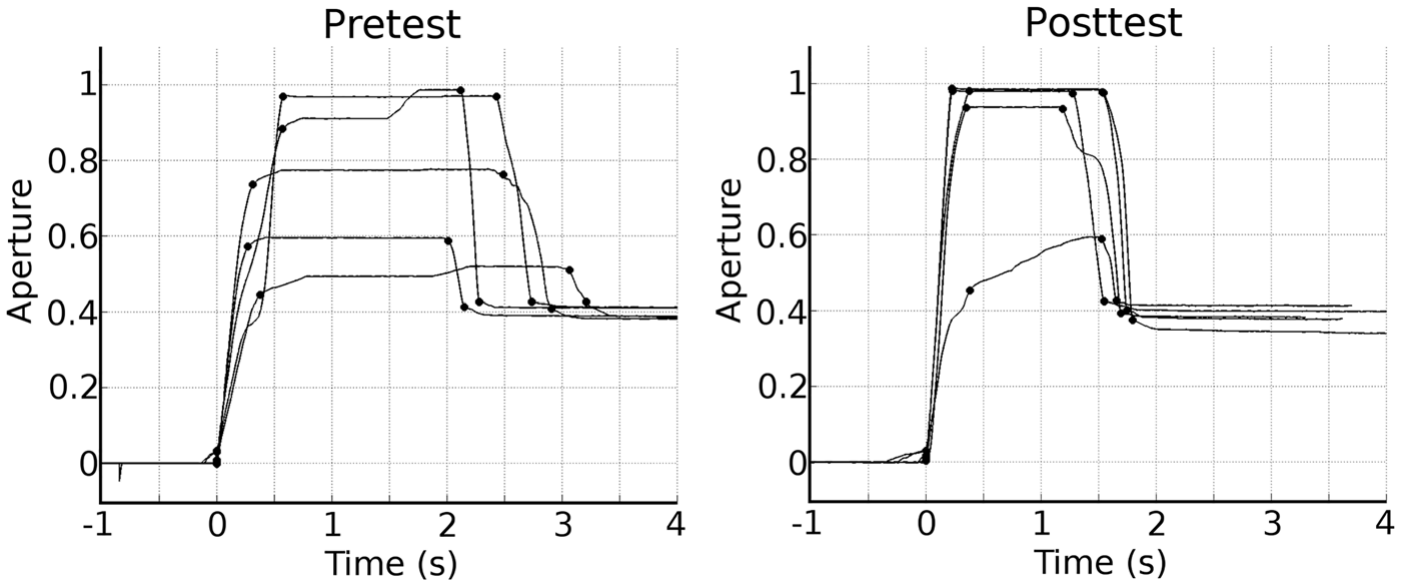
Prosthesis hand aperture. Representative example of the hand aperture during the pretest (left figure) and posttest (right figure) during grasping a medium size cylinder. Both examples are from the same participant. This participant was assigned to the Breakout-EMG group, however the example is equally representative for the Control group. The time (s) is shown on the x-axis, the normalized aperture on the y-axis. A change in aperture of 0.1 corresponds to a change of ~1 cm in distance between thumb and index finger. The lines represent five trials of grasping a (medium) cylinder. The four round markers on each line represent (from left to right) the start of the opening, the end of the opening, the start of the closing and the end of the closing of the hand.

A typical example of the aperture of the myoelectric over time is shown in Fig 6. Due to a technical problem one of the participants in the control group could not complete the pretest prosthesis task and was excluded from further analysis.

First, we looked at the maximum hand opening. The (normalized) maximum hand opening is shown in Table 2. Statistical analysis revealed a small significant effect for cylinder (*F*(1,25) = 9.67, *p* < .001, *η*^*2*^_*G*_ = .05). There was no significant main effect for Test, and there was no significant interaction effect Test x Group.

**Table 2:** Results of the prosthesis task. Mean (and standard error of the mean in brackets) of the normalized maximum hand opening (MHO) (0–1; 0 being closed and 1 being fully opened), as well as of the standard deviation of the normalized maximum hand opening (SD-MHO), and of the duration of the plateau phase (s) for the small and the medium cylinder for both groups on the pretest and the posttest.

	Pretest	Posttest
**MHO, small**		
**Breakout-EMG**	0.95 (0.03)	0.96 (0.02)
**Control**	0.87 (0.05)	0.91 (0.05)
**MHO, medium**		
**Breakout-EMG**	0.98 (0.01)	0.99 (0.01)
**Control**	0.97 (0.01)	0.94 (0.05)
**SD-MHO, small**		
**Breakout-EMG**	0.04 (0.02)	0.04 (0.01)
**Control**	0.06 (0.03)	0.07 (0.03)
**SD-MHO, medium**		
**Breakout-EMG**	0.02 (0.01)	0.01 (0.01)
**Control**	0.03 (0.02)	0.02 (0.01)
**Plateau phase, small**		
**Breakout-EMG**	1.71 (0.18)	1.81 (0.28)
**Control**	1.74 (0.16)	1.51 (0.13)
**Plateau phase, medium**		
**Breakout-EMG**	1.67 (0.18)	1.49 (0.16)
**Control**	1.69 (0.14)	1.35 (0.13)

Adaptation of the hand aperture to the size of the cylinder could also be expected through a three-way interaction of Test x Group x Cylinder. That is, the difference in the hand aperture between cylinders is expected to increase over time for the Breakout-EMG groups more than for the Controls. Analysis however revealed no significant three-way interaction effect.

The duration of the plateau phase can also be seen in Table 2. Analysis revealed a small significant effect for cylinder (*F*(1,25) = 6.74, *p* = .016, *η*^*2*^_*G*_ = .04). There was no significant main effect for Test, nor were there any significant interaction effects.

The standard deviation of the normalized maximum hand opening is also shown in Table 2. As one of the participants in the control group had only one correct grasp of the small cylinder during the pretest, the standard deviation in MHO could not be established in this case. We excluded this participant from analysis. Analysis revealed a significant effect for cylinder (*F*(1,24) = 9.52, *p* < .001, *η*^*2*^_*G*_ = .14). There were no other significant effects.

## Discussion

In this study we tested whether a simple myogame that conforms to the specifications of the current generation of myogames (i) can be learned, and if so (ii) what changes in the myosignal may account for such in-game learning. Finally, we tested (iii) whether in-game improvement would transfer to a prosthesis task conforming to the settings typically used in clinical practice. Our results showed that performance on playing the Breakout-EMG game improved significantly in comparison to controls. Moreover, we showed that the increase in in-game performance was associated with an increase in the EMG-ball coupling. When compared to the control group however, we found no indications of transfer of this skill to a prosthesis task. That is, the participants learned to adjust the EMG signal they generated specifically to the requirements of the gaming task.

The main aim of this study was to determine what learning and transfer effects can be expected from training with the current generation of myogames. Therefore we aimed to maximize our chances of finding transfer. To do so, we chose a “sham” control group to control for testing-effects, for the amount of training, and for motivational aspects such as novelty effects or the effect of being part of an experiment (a Hawthorne effect). We did not choose a comparable myoelectric interface with comparable muscular involvement for the controls. With respect to transfer, this meant that all positive effects of training the game on prosthesis use should have shown up in the post test performance. Thus, in our opinion, the current set-up maximized chances of finding transfer to our prosthesis-task. Consequently however, if we had found transfer, we would not have been able to pin-point its likely origin. Combining the current results with previous transfer effects [12] however, creating more subtle control conditions will be an interesting next step in order to tease out how different aspects of a game can influence transfer.

As a step in improving the design of myogames for prosthesis use, the current study aimed to provide an evaluation of current practices: it stayed close to both the settings and designs of myogaming research and to settings clinically used in prosthesis fitting. We thus designed a basic game much like those currently used, a game that was fun to play and easy to control by myosignals. We trained participants to play this game using the same muscles as they had to use for handling a prosthesis simulator. The EMG signals were furthermore proportionally related to the speed of the end-effector, just as in a prosthesis task. Thus we followed the same logic as earlier studies using myogames [6,8], but extended this to include a transfer test. If improvement in prosthetic control was, for example, based on isolating muscular activity, the repetitive generation of EMG signals or on re-calibrating the acquired EMG control to a new range (see also [27]), we should have found transfer to our prosthesis task. Our results however, corroborate earlier findings [12] and imply that creating a game that transfers effectively to ADL, may not be that easy.

In the end, myogame training should make the transfer to starting to practice with an actual prosthesis easier. It might therefore have its biggest role early in the rehabilitation process (i.e., in the pre prosthetic phase), when for example neural plasticity is high but wound healing prohibits the use of a prosthetic device (see [2;11]). To facilitate transfer, our study points to several design features that deserve scrutiny in future myogame development. First, Breakout-EMG required less activation to play than did the myoelectric hand (i.e. 20% MVC, which is ~80% “comfortable contraction”, see [1,10]). In as far as the calibration to MVC is reported, this is a common design choice that is aimed at preventing fatigue during training [1,8,10,28]. In accordance with clinical practice [29], the myoelectric hand was however calibrated so that the maximum opening speed required the MVC sustainable for 2 seconds. Although it was recently shown that aligning the EMG intensity required for in-game performance with actual prosthesis use is insufficient for allowing transfer [12], this does not preclude the possibility that it could create favorable conditions for transfer to occur. Thus it seems that future myogames should aim to determine the effects of these settings.

Second, as any myoelectric prosthesis, our prosthesis simulator had a time delay between generating the myosignal and the change in aperture. Such a delay was not present in the game as this would have made our game unplayable. As our grasping task was self-paced, timing the EMG signal was much less critical than in Breakout-EMG. It has been shown in a controlled pre-posttest design that simulating this delay is not sufficient to allow for transfer [12]. Nonetheless, it may still be beneficial to accommodate for a delay parameter in a future game design. To do so however, we need better estimates of the movement characteristics of currently available prostheses in relation to the generated EMG signals. To our knowledge, such estimates are not currently available. Future research should aim to establish these estimates and determine their exact effects on transfer.

An interesting aspect of our current study is our finding on in-game learning. This may help to guide ideas to improve myogaming for prosthesis use. The development of a strengthened coupling between the generated EMG signal and the game implies that during the game a very task-specific adaptation of the myosignal occurred; participants coupled their EMG directly to the required distance to make the paddle move in order to intercept the ball. This may indicate that when learning a myoelectric skill, it is not the myosignal that is being controlled as such, nor is control limited to the relation between the signal and the movement of the end-effector. What is being controlled might be the myosignal relative to goal-relevant information in the task (see also e.g. [12,14,30,31]). Our in-game learning effects thus add to the previous effect study [12] by suggesting that transfer is enabled in so far as the myosignal can be coordinated to the similar goal-relevant information across tasks.

An important limitation of our current set-up was our use of able bodied participants controlling a prosthesis simulator. So far, myogame research has not shown much empirical evidence for their benefit (see e.g. [1,3,4,6–10], but see [12]). The current designs therefore do not yet warrant testing for motor learning effects on patient groups. Simulators have been used before to approximate prosthesis use and it appeared that kinematic performances is comparable to performances with real upper limb prostheses [22]. The advantage of using these methods is that the small population of persons with upper limb amputations will not be bothered with research that does not lead to clinically useful results. Generalization to clinical populations that are already using myogames should however be handled with caution–especially since myogames might also be used for rehabilitation goals other than motor learning. It should also be noted that we cannot rule out that our training period was too short for transfer to occur. Although previous research that used the same amount of training was successful in this respect [12], and although we did find in-game learning effects, transfer of this particular game might require more extensive training time.

## Conclusions

Although myogames are becoming an integral part of rehabilitation, designing a game that actually transfers to ADL is far from trivial. The marked improvement in myogame performance does not transfer to a prosthesis task. We have thus shown the need to explicitly design myogames for transfer to daily life and hope to have put some new design considerations on the map. By providing an evaluation of the transfer effect of the current generation of myogames, this study can provide a starting point for developing myogames that successfully transfer to activities of daily life.

## Supporting Information

S1 File Data file. (XLSX)
